# A characterization for fuzzy strong cut vertices and fuzzy strong cut edges

**DOI:** 10.1038/s41598-024-66274-9

**Published:** 2024-07-04

**Authors:** Junye Ma, Lijing Shen, Lin Li

**Affiliations:** 1 School of Applied Science, Taiyuan University of Science and Technology, Taiyuan, 030024 People’s Republic of China; 2 School of Mechanical Engineering, Taiyuan University of Science and Technology, Taiyuan, 030024 People’s Republic of China

**Keywords:** Mathematics and computing, Applied mathematics, Computational science

## Abstract

Cut vertices and cut edges are valuable for analyzing connectivity problems in classical graph theory. However, they cannot deal with certain fuzzy problems. In order to solve this problem, this paper introduces the definitions of fuzzy strong cut vertices and fuzzy strong cut edges, and characterizes fuzzy strong cut vertices and fuzzy strong cut edges in fuzzy trees, complete fuzzy graphs, and fuzzy cycles. Finally, practical applications verify the effectiveness of the theory in network stability analysis.

## Introduction

### Research background

The concept of fuzzy graphs can be traced back to the pioneering research conducted by Rosenfeld^1^, which had a significant impact. Rosenfeld’s groundbreaking contributions have served as inspiration for numerous scholars, prompting them to explore the theory of fuzzy graphs more extensively and to engage in additional research within this field. Notable contributions were made by Nair^2^ in characterizing fuzzy bridges. The definition of strong edges in fuzzy graphs was introduced by Bhutani and Rosenfeld^3^, who subsequently delved into their correlation with fuzzy bridges. Sunitha and Vijayakumar^4^ introduced the definition of fuzzy blocks. Mathew and Sunitha^5^ established a sufficient condition that determines whether a fuzzy graph can be classified as a fuzzy block. Mathew et al.^6^ introduced the concepts of connectivity-transitive fuzzy graphs and cyclically-transitive fuzzy graphs to investigate the properties of fuzzy blocks. Furthermore, four different characterizations of fuzzy blocks were obtained by them. Akram et al.^7^ introduced the definition of m-polar fuzzy strength sequence of vertices whose exploration was specifically carried out in the context of complete m-polar fuzzy graphs. The relationship between fuzzy independent dominating sets and fuzzy minimal dominating sets was studied by Nagoorgani et al.^8^. In 2021, Ma et al.^9^ gave the definition of fuzzy edge connectivity and fuzzy local edge connectivity and investigated their related properties. In order to explore the properties of the nonlinear model of COVID-19 propagation, the idea of fuzzy mapping was brought by Panda et al.^10^ to analyze the existence of a solution. Within the available literature, there exists a wealth of related works and applications in the domain of fuzzy graph theory, offering abundant avenues for further investigation^11–15^.

### Motivation

Spacious and high-flow routes in a road transportation network are important for the transportation of goods between hubs. However, the concept of paths in classical graph theory cannot depict the problem. Considering that many research papers^16–19^ have been devoted to exploring the relevant properties and applications of paths in fuzzy graphs, inspired by these previous studies, we set out to investigate the strongest strong paths. In this paper, we introduce the definitions of fuzzy strong cut vertices and fuzzy strong cut edges. By investigating their interconnection properties, an approach is proposed to identify critical hubs and routes in a transportation network so as to ensure the stability of the transportation system.

### Framework of this study

In this paper, Section 2 establishes the symbols and terminologies used in this context. In Section 3, we investigate the related properties of fuzzy strong cut vertices and fuzzy strong cut edges in fuzzy trees, complete fuzzy graphs, and fuzzy cycles. Furthermore, the characterization of the relationship between fuzzy strong cut vertices and fuzzy strong cut edges is presented. Section 4 demonstrates the relevant applications of fuzzy strong cut vertices and fuzzy strong cut edges in a transportation network, which reflect the advantages of fuzzy strong cut vertices and fuzzy strong cut edges in determining network connectivity.

## Preliminaries

Various symbols and terminologies used in this paper are explained below, and related definitions were provided by Mathew et al.^20^.

$$\begin{aligned} \begin{array}{ll} G=(V, \sigma , \mu ) &{} \text {{a finite and connected undirected fuzzy graph without loops}} \\ V &{} \text {the set of all vertices in } G\\ E(G) &{} \text {the set of all edges in } G\\ \sigma ^{*}=\{a\in V\mid \sigma (a)> 0\} &{} \text {the set of vertices whose membership value is greater than 0 in } G \\ \mu ^{*}=\{(a, b)\in V\times V\mid \mu (a, b)> 0\}&{} \text {the set of edges whose membership value is greater than 0 in } G \\ s(P) &{} \text {the strength of a path } P\\ CONN_{G}(a, b) &{} \text {the strength of connectedness between } a \text { and } b \text { in } G\\ \end{array} \end{aligned}$$

## Fuzzy strong cut vertices and fuzzy strong cut edges

In this section, the definitions of fuzzy strong cut vertices and fuzzy strong cut edges are first introduced. Following that, these elements are characterized within the domains of fuzzy trees, complete fuzzy graphs, and fuzzy cycles. To conclude, an in-depth investigation is conducted into the intricate interrelationship between fuzzy strong cut vertices and fuzzy strong cut edges.

### Definition 1

^20^ Let $G=(V,\sigma ,\mu )$ be a fuzzy graph and $u,v\in \sigma ^{*}$, *P* be a strongest $u-v$ path in *G*. If *P* is a strong path, then *P* is called a fuzzy strongest strong $u-v$ path.

### Definition 2

Let $G=(V,\sigma ,\mu )$ be a fuzzy graph and $S\subseteq V$. If there exist fuzzy strongest strong $u-v$ paths in *G* for some pair of vertices $u,v\in \sigma ^{*} \backslash S$, but there exists no fuzzy strongest strong $u-v$ path in $G-S$, then *S* is called a fuzzy strong vertex cut of *G*. If there is only one vertex *w* in *S*, then *w* is called a fuzzy strong cut vertex of *G*.

### Definition 3

Let $G=(V,\sigma ,\mu )$ be a fuzzy graph and $D\subseteq E(G)$. If there exist fuzzy strongest strong $u-v$ paths in *G* for some pair of vertices $u,v\in \sigma ^{*}$, but there exists no fuzzy strongest strong $u-v$ path in $G-D$, then *D* is called a fuzzy strong edge cut of *G*. If there is only one edge *xy* in *D*, then *xy* is called a fuzzy strong cut edge of *G*.

In this paper, we assume that $\sigma (x)=1$ for $x\in \sigma ^{*}$ in all examples of fuzzy graph $G=(V,\sigma ,\mu )$, for convenience.

### Example 1

**Figure 1:**
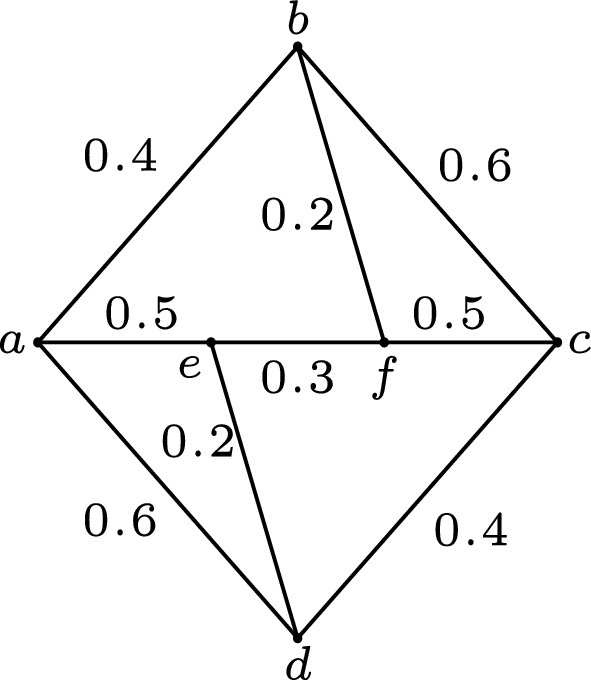
Fuzzy graph in Example 1.

Let $G=(V,\sigma ,\mu )$ be a fuzzy graph with $V=\{a, b, c, d, e, f\}$, $\mu (b, f)=\mu (d, e)=0.2$, $\mu (e, f)=0.3$, $\mu (a, b)=\mu (c, d)=0.4$, $\mu (a, e)=\mu (f, c)=0.5$, $\mu (b, c)=\mu (a, d)=0.6$. According to the definition of fuzzy strongest strong paths, we can deduce that $a\rightarrow b\rightarrow c$ and $a\rightarrow d\rightarrow c$ are two fuzzy strongest strong $a-c$ paths in *G*, $b\rightarrow a\rightarrow e$ and $b\rightarrow c\rightarrow d\rightarrow a\rightarrow e$ are two fuzzy strongest strong $b-e$ paths in *G*. Let $S=\{b, d\}$, $D=\{ab, cd\}$. Since there exists no fuzzy strongest strong $a-c$ path in $G-S$ and there exists no fuzzy strongest strong $a-c$ path in $G-D$, *S* is a fuzzy strong vertex cut of *G* and *D* is a fuzzy strong edge cut of *G*. Similarly, since there exists no fuzzy strongest strong $b-e$ path in $G-\{a\}$ and there exists no fuzzy strongest strong $b-e$ path in $G-\{ae\}$, the vertex *a* is a fuzzy strong cut vertex of *G* and the edge *ae* is a fuzzy strong cut edge of *G*. See Fig. 1 for more details.

### Lemma 1

^21^ Let $G=(V,\sigma ,\mu )$ be a fuzzy tree with *H* its unique maximum spanning tree and $uv\in \mu ^{*}$. Then *uv* is $\alpha$-strong if and only if *uv* is an edge in *H*.

### Lemma 2

^3^ Let $G=(V,\sigma ,\mu )$ be a fuzzy tree and $u,v\in \sigma ^{*}$. If *P* is a strong $u-v$ path in *G*, then *P* is a strongest $u-v$ path in *G*.

### Lemma 3

^3^ Let $G=(V,\sigma ,\mu )$ be a fuzzy graph, then *G* is a fuzzy tree if and only if there is a unique strong path in *G* between any two vertices of *G*.

### Lemma 4

^21^ Let $G=(V,\sigma ,\mu )$ be a fuzzy graph, then *G* is a fuzzy tree if and only if it has no $\beta$-strong edges.

### Theorem 1

Let $G=(V,\sigma ,\mu )$ be a fuzzy tree with *H* its unique maximum spanning tree, $w\in \sigma ^{*}$. Then *w* is a fuzzy strong cut vertex of *G* if and only if *w* is an internal vertex of *H*.

### Proof

$(\Rightarrow )$ Suppose that *w* is a fuzzy strong cut vertex of *G*. Since *G* is a fuzzy tree, there exists a unique fuzzy strongest strong path in *G* between any two vertices of *G*. From the definition of fuzzy strong cut vertices, we can get that there exists no fuzzy strongest strong $u-v$ path in $G-\{w\}$ for some pair of vertices $u,v\in \sigma ^{*}$. Thus *w* is in the unique fuzzy strongest strong $u-v$ path. Let *P* be the fuzzy strongest strong $u-v$ path in *G*, *xw* and *wy* be two edges in *P*. In view of Lemmas 1 and 4, all the edges in *P* are the edges of *H*. So $xw, wy \in E(H)$. Thus *w* is an internal vertex of *H*.

$(\Leftarrow )$ Suppose that *w* is an internal vertex of *H*. Then *w* is the common vertex of at least two edges in *H*. Let *xw* and *wy* be the two edges in *H*. By Lemma 1, we deduce that *xw* and *wy* are two $\alpha$-strong edges in *G*. Thus $x\rightarrow w\rightarrow y$ is a strong $x-y$ path in *G*. On account of Lemmas 2 and 3, $x\rightarrow w\rightarrow y$ is a unique fuzzy strongest strong $x-y$ path in *G*. Since there exists no fuzzy strongest strong $x-y$ path in $G-\{w\}$, *w* is a fuzzy strong cut vertex of *G*. $\square$

### Theorem 2

Let $G=(V,\sigma ,\mu )$ be a fuzzy tree with *H* its unique maximum spanning tree, $uv\in \mu ^{*}$. Then *uv* is a fuzzy strong cut edge of *G* if and only if *uv* is an edge in *H*.

### Proof

$(\Rightarrow )$ Suppose that *uv* is a fuzzy strong cut edge of *G*. Since *G* is a fuzzy tree, there exists a unique fuzzy strongest strong path in *G* between any two vertices of *G*. According to the definition of fuzzy strong cut edges, there exists no fuzzy strongest strong $x-y$ path in $G-\{uv\}$ for some pair of vertices $x,y\in \sigma ^{*}$. Hence, *uv* must be in the unique fuzzy strongest strong $x-y$ path *P*. On account of Lemmas 1 and 4, all the edges in *P* are the edges in *H*. Thus *uv* is an edge in *H*.

$(\Leftarrow )$ Suppose that *uv* is an edge in *H*, then *uv* is $\alpha$-strong by Lemma 1. $u\rightarrow v$ is a unique fuzzy strongest strong $u-v$ path in view of Lemma 3. This shows that there exists no fuzzy strongest strong $u-v$ path in $G-\{uv\}$. Thus, *uv* is a fuzzy strong cut edge of *G*. $\square$

### Lemma 5

^22^ If $G=(V,\sigma ,\mu )$ is a complete fuzzy graph, then $\mu ^{\infty }(u,v)=\mu (u,v)$ for any edge $uv\in \mu ^{*}$.

### Lemma 6

^21^ If $G=(V,\sigma ,\mu )$ is a complete fuzzy graph, then it has no $\delta -$edges.

### Theorem 3

If $G=(V,\sigma ,\mu )$ is a complete fuzzy graph, then there exists no fuzzy strong cut vertex in *G*.

### Proof

Let $w\in \sigma ^{*}$. Assume that *w* is a fuzzy strong cut vertex of *G*. In view of Lemmas 5 and 6, $x\rightarrow y$ is a strongest strong $x-y$ path in *G* for any two vertices $x,y\in \sigma$. According to the definition of fuzzy strong cut vertices, there exists no fuzzy strongest strong $u-v$ path in $G-\{w\}$ for some pair of vertices $u,v\in \sigma ^{*}$. Thus *w* must be in every fuzzy strongest strong $u-v$ path. However, this is not possible that *w* is in the path $u\rightarrow v$. Therefore, there exists no fuzzy strong cut vertex in *G*. $\square$

### Theorem 4

Let $G=(V,\sigma ,\mu )$ be a complete fuzzy graph with the vertex set $V=\{x_{1}, x_{2}, \ldots , x_{n}\}$. Then *G* has a fuzzy strong cut edge if and only if there exists an increasing sequence $\{\sigma (x_{1}), \sigma (x_{2}), \ldots , \sigma (x_{n})\}$ such that $\sigma (x_{n-2})<\sigma (x_{n-1})\le \sigma (x_{n})$. Also the edge $x_{n-1}x_{n}$ is the fuzzy strong cut edge of *G*.

### Proof

$(\Rightarrow )$ Suppose that *G* has a fuzzy strong cut edge *uv*. Without loss of generality, let $\sigma (u)\le \sigma (v)$. Since *G* is a complete fuzzy graph, $\mu (u,v)=\sigma (u)\wedge \sigma (v)=\sigma (u)$. According to Lemmas 5 and 6, for any two vertices *x* and *y*, $x\rightarrow y$ is a fuzzy strongest strong $x-y$ path in *G*. Thus, $u\rightarrow v$ is a fuzzy strongest strong $u-v$ path in *G* with the strength $\sigma (u)$. Next we claim that $\sigma (u)>\sigma (w)$ for all $w\ne v$. Assume to the contrary that there exists a vertex $w\ne v$ satisfying that $\sigma (u)\le \sigma (w)$. Then $u\rightarrow w\rightarrow v$ is a fuzzy strongest strong $u-v$ path in *G* with the strength $\sigma (u)$. This shows that there still exist fuzzy strongest strong $u-v$ paths for $u,v\in \sigma ^{*}$, after deleting the edge *uv* from *G*. For any two vertices *r*, *t* satisfying $r\ne u$ and $r\ne v$, there exists a fuzzy strongest strong $r-t$ path $r\rightarrow t$ after deleting the edge *uv* from *G*. This is a contradiction that *uv* is a fuzzy strong cut edge of *G*. Thus $\sigma (u)>\sigma (w)$ for all $w\ne v$, we get the conclusion.

$(\Leftarrow )$ Suppose that there exists an increasing sequence $\{\sigma (x_{1}), \sigma (x_{2}), \sigma (x_{3}), \ldots , \sigma (x_{n})\}$ such that $\sigma (x_{n-2})<\sigma (x_{n-1})\le \sigma (x_{n})$. We claim that the edge $x_{n-1}x_{n}$ is a fuzzy strong cut edge of *G*. According to Lemmas 5 and 6, $x_{n-1}\rightarrow x_{n}$ is a fuzzy strongest strong $x_{n-1}-x_{n}$ path in *G* with the strength $\sigma (x_{n-1})$. In view of the hypothesis, $x_{n-1}\rightarrow x_{n}$ is a unique fuzzy strongest strong $x_{n-1}-x_{n}$ path in *G*. Thus there exists no fuzzy strongest strong $x_{n-1}-x_{n}$ path in $G-\{x_{n-1}x_{n}\}$. So the edge $x_{n-1}x_{n}$ is a fuzzy strong cut edge of *G*. $\square$

Let $G=(V,\sigma ,\mu )$ be a fuzzy cycle and $uv\in \mu ^{*}$. On account of the definition of fuzzy cycles, *uv* is the weakest edge in *G* if and only if *uv* is $\beta$-strong; *uv* is not the weakest edge in *G* if and only if *uv* is $\alpha$-strong.

### Theorem 5

Let $G=(V,\sigma ,\mu )$ be a fuzzy cycle, $w\in \sigma ^{*}$. Then *w* is a fuzzy strong cut vertex of *G* if and only if there exist two $\alpha$-strong edges incident with *w* in *G*.

### Proof

$(\Rightarrow )$ Suppose that *w* is a fuzzy strong cut vertex of *G*. According to the definition of fuzzy strong cut vertices, there exists no fuzzy strongest strong $u-v$ path in $G-\{w\}$ for some pair of vertices $u,v\in \sigma ^{*}$. Since *G* is a fuzzy cycle, *w* must be in a unique fuzzy strongest strong $u-v$ path *P*. Furthermore, all the edges in *P* are $\alpha$-strong. Thus, there exist two $\alpha$-strong edges incident with *w* in *G*.

$(\Leftarrow )$ Suppose that there exist two $\alpha$-strong edges incident with *w* in *G*. Let *xw* and *wy* be the two edges in *G*. Thus $x\rightarrow w\rightarrow y$ is a unique fuzzy strongest strong $x-y$ path in *G*. Since there exists no fuzzy strongest strong $x-y$ path in $G-\{w\}$, *w* is a fuzzy strong cut vertex of *G*. $\square$

### Theorem 6

Let $G=(V,\sigma ,\mu )$ be a fuzzy cycle and $uv\in \mu ^{*}$. Then *uv* is a fuzzy strong cut edge of *G* if and only if *uv* is not the weakest edge in *G*.

### Proof

$(\Rightarrow )$ Suppose that *uv* is a fuzzy strong cut edge of *G*. According to the definition of fuzzy strong cut edges, there exists no fuzzy strongest strong $x-y$ path in $G-\{uv\}$ for some pair of vertices $x,y\in \sigma ^{*}$. Thus *uv* must be in every fuzzy strongest strong $x-y$ path. Since *G* is a fuzzy cycle, the fuzzy strongest strong $x-y$ path in *G* is unique. Let *P* be the fuzzy strongest strong $x-y$ path in *G*. According to the definition of fuzzy cycles, the edge in *G* is either $\alpha$-strong or $\beta$-strong. We claim that *uv* is $\alpha$-strong. Assume to the contrary that *uv* is $\beta$-strong. By the definition of fuzzy cycles, an edge in *G* is the weakest edge in *G* if and only if the edge is $\beta$-strong, an edge in *G* is not the weakest edge in *G* if and only if the edge is $\alpha$-strong. So *uv* is the weakest edge in *G*. Hence, the strength of *P* is equal to $\mu (u,v)$, and there exist two fuzzy strongest strong $x-y$ paths in *G*, which is a contradiction that the fuzzy strongest strong $x-y$ path in *G* is unique. Thus, *uv* is $\alpha$-strong and *uv* is not the weakest edge in *G*.

$(\Leftarrow )$ Suppose that *uv* is not the weakest edge in *G*. In view of the definition of fuzzy cycles, *uv* is $\alpha -$strong. Thus, $u\rightarrow v$ is a unique fuzzy strongest strong $u-v$ path in *G*. Hence, there exists no fuzzy strongest strong $u-v$ path in $G-\{uv\}$. This shows that *uv* is a fuzzy strong cut edge of *G*. $\square$

Sunitha and Vijayakumar^23^ delved into the intricate relationship between fuzzy cut vertices and fuzzy bridges, uncovering fascinating insights. In the following, we similarly explore the association between fuzzy strong cut vertices and fuzzy strong cut edges. By leveraging Theorems 1, 2, 5, and 6, this relationship will be first characterized within the framework of fuzzy trees and fuzzy cycles. Furthermore, more general cases are discussed.

### Theorem 7

Let $G=(V,\sigma ,\mu )$ be a fuzzy tree, $w\in \sigma ^{*}$. Then *w* is a fuzzy strong cut vertex of *G* if and only if *w* is the common vertex of at least two fuzzy strong cut edges of *G*.

### Theorem 8

Let $G=(V,\sigma ,\mu )$ be a fuzzy cycle, $w\in \sigma ^{*}$. Then *w* is a fuzzy strong cut vertex of *G* if and only if *w* is the common vertex of two fuzzy strong cut edges of *G*.

### Theorem 9

Let $G=(V,\sigma ,\mu )$ be a fuzzy graph and $xw,wy\in E(G)$. If *xw* and *wy* are two fuzzy strong cut edges of *G*, then *w* is a fuzzy strong cut vertex of *G*.

### Proof

Suppose that *xw* and *wy* are two fuzzy strong cut edges of *G*. According to the definition of fuzzy strong cut edges, there exist fuzzy strongest strong $u_{1}-v_{1}$ paths in *G* for some pair of vertices $u_{1},v_{1}\in \sigma ^{*}$, but there exists no fuzzy strongest strong $u_{1}-v_{1}$ path in $G-\{xw\}$. Thus *xw* must be in every fuzzy strongest strong $u_{1}-v_{1}$ path. Let *P* be a fuzzy strongest strong $u_{1}-v_{1}$ path in *G*. We claim that *xw* is an $\alpha$-strong edge in *G*. Assume to the contrary that *xw* is $\beta$-strong. Then there exists a strong $x-w$ path $P_{1}$ with the strength $\mu (x,w)$ which is different from $x\rightarrow w$ in *G*. Replacing *xw* with $P_{1}$ in this path *P*, we will get a new fuzzy strongest strong $u_{1}-v_{1}$ path, which is a contradiction that *xw* is a fuzzy strong cut edge of *G*. Thus *xw* is $\alpha$-strong. Similarly, we can prove that *wy* is an $\alpha$-strong edge in *G*. Let’s talk about two cases. Case 1: $x\rightarrow w\rightarrow y$ is a unique fuzzy strongest strong $x-y$ path in *G*. So there exists no fuzzy strongest strong $x-y$ path in $G-\{w\}$. Therefore, *w* is a fuzzy strong cut vertex of *G*. Case 2: There exist other fuzzy strongest strong $x-y$ paths in *G*. We claim that *w* is in every fuzzy strongest strong $x-y$ path. Assume to the contrary that there exists a fuzzy strongest strong $x-y$ path $P^{'}$ in *G* which does not contain the vertex *w*. Without loss of generality, let $\mu (x,w)\le \mu (w,y)$, then $s(P^{'})=CONN_G(x,y)=\mu (x,w)$. The combination of this path $P^{'}$ and this edge *wy* forms a new $x-w$ path with the strength $\mu (x,w)$, which is a contradiction that *xw* is $\alpha$-strong. So *w* is in every fuzzy strongest strong $x-y$ path. Thus there exists no fuzzy strongest strong $x-y$ path in $G-\{w\}$. Therefore, *w* is a fuzzy strong cut vertex of *G*. $\square$

### Remark 1

The converse of Theorem 9 is not true. Let $G=(V,\sigma ,\mu )$ be a fuzzy graph and *w* be a fuzzy strong cut vertex of *G*. Then *w* may be not the common vertex of two fuzzy strong cut edges of *G*.

### Example 2

**Figure 2:**
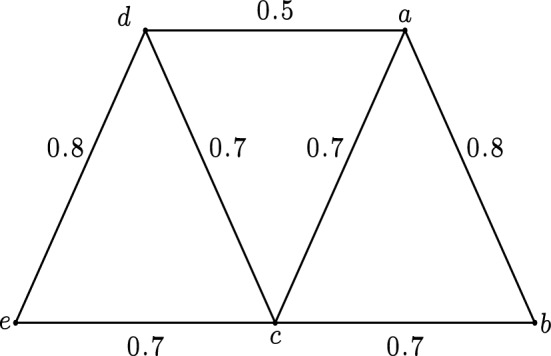
Fuzzy graph in Example 2.

(**Justification of Remark** 1) Let $G=(V,\sigma ,\mu )$ be a fuzzy graph with $V=\{a, b, c, d, e\}$, $\mu (a, d)=0.5$, $\mu (a, c)=\mu (c, b)=\mu (c, d)=\mu (c, e)=0.7$, $\mu (a, b)=\mu (e,d)=0.8$. Applying the definition of fuzzy strongest strong paths, we can get that $e\rightarrow c\rightarrow b$ and $e\rightarrow d\rightarrow c\rightarrow a\rightarrow b$ are two fuzzy strongest strong $e-b$ paths in *G*. Since there exists no fuzzy strongest strong $e-b$ path in $G-\{c\}$, the vertex *c* is a fuzzy strong cut vertex of *G*. Similarly, we can easily prove that the edges *ca*, *cb*, *cd*, *ce* are not fuzzy strong cut edges of *G*. See Fig. 2 for more details.

### Remark 2

Let $G=(V,\sigma ,\mu )$ be a fuzzy graph and $xy\in E(G)$. If the edge *xy* is a fuzzy strong cut edge of *G*, then *xy* may be the weakest edge in *G*.

### Example 3

**Figure 3:**
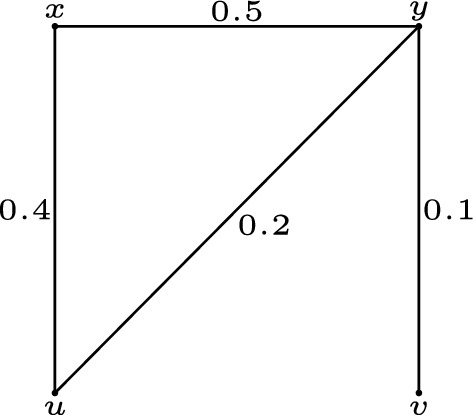
Fuzzy graph in Example 3.

(**Justification of Remark** 2) Let $G=(V,\sigma ,\mu )$ be a fuzzy graph with $V=\{x, y, u, v\}$, $\mu (x, y)=0.5$, $\mu (y, u)=0.2$, $\mu (y, v)=0.1$, $\mu (x, u)=0.4$. Using the definition of fuzzy strongest strong paths, we deduce that $u\rightarrow x\rightarrow y\rightarrow v$ is a fuzzy strongest strong $u-v$ path in *G*. As there exists no fuzzy strongest strong $u-v$ path in $G-\{yv\}$, the edge *yv* is a fuzzy strong cut edge of *G*. However, *yv* is the weakest edge in *G*. See Fig. 3 for more details.

### Theorem 10

Let $G=(V,\sigma ,\mu )$ be a fuzzy graph and $xy\in E(G)$. Then *xy* is a fuzzy strong cut edge of *G* if and only if *xy* is $\alpha$-strong.

### Proof

$(\Rightarrow )$ Suppose that *xy* is a fuzzy strong cut edge of *G*. According to the definition of fuzzy strong cut edges, there exist fuzzy strongest strong $u-v$ paths in *G* for some pair of vertices *u*, *v*, but there exists no fuzzy strongest strong $u-v$ path in $G-\{xy\}$. Thus *xy* must be in every fuzzy strongest strong $u-v$ path. Let *P* be a fuzzy strongest strong $u-v$ path in *G*. We claim that *xy* is an $\alpha$-strong edge in *G*. Assume to the contrary that *xy* is $\beta$-strong. Then there exists a strong $x-y$ path $P^{'}$ with the strength $\mu (x,y)$ which is different from $x\rightarrow y$ in *G*. Replacing *xy* with $P^{'}$ in this path *P*, we will get a new fuzzy strongest strong $u-v$ path, which is a contradiction that *xy* is a fuzzy strong cut edge of *G*. Thus *xy* is $\alpha$-strong.

$(\Leftarrow )$ Suppose that *xy* is $\alpha$-strong. Then $x \rightarrow y$ is a unique fuzzy strongest strong $x-y$ path in *G*. So there exists no fuzzy strongest strong $x-y$ path in $G-\{xy\}$. Therefore, *xy* is a fuzzy strong cut edge of *G*. $\square$

### Remark 3

According to Theorem 10, the problem of searching for all the fuzzy strong cut edges in a fuzzy graph is equivalent to searching for all $\alpha$-strong edges, which can be obtained by Mathew’s Algorithm^21^. The time complexity of this algorithm is $O(n^{2})$.

With the help of Mathew’s algorithm^21^ and Banerjee’s Algorithm^24^, all the fuzzy strong cut vertices of a fuzzy graph can be identified, see Algorithm 1 for detailed steps.

**Figure:**
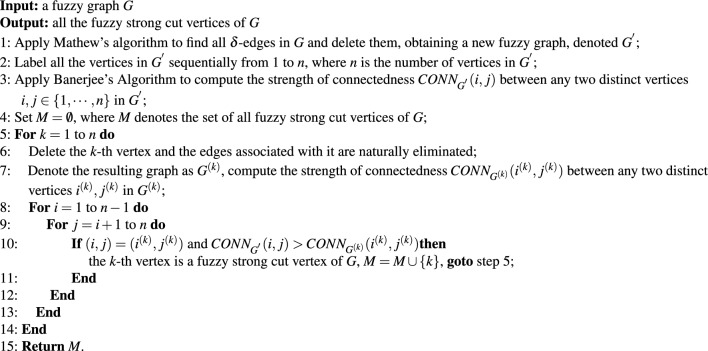
Algorithm 1 Searching for all the fuzzy strong cut vertices of a fuzzy graph

In Algorithm 1, the time complexity of Mathew’s algorithm and Banerjee’s algorithm both are O($n^2$). Step 5 requires O(*n*) time, since the number of vertices is *n*. Step 8 and Step 9 involve two nested for loops with time complexity O$(n^2)$. Considering that the algorithm involves a time complexity of O$(n^2)$ in the outer for loop starting from step 5, the total time complexity of Algorithm 1 is O$(n^3)$.

### Remark 4

In the field of directed fuzzy graphs, the concepts of fuzzy strong cut vertices and fuzzy strong cut edges are similar to their counterparts in undirected fuzzy graphs. Therefore, these concepts introduced in this paper can be easily extended to directed fuzzy graphs as well.

## The applications of fuzzy strong cut vertices and fuzzy strong cut edges

Road transport network refers to the transport network composed of road transport routes and transport hubs within a certain area in accordance with certain principles and requirements. Road transport routes are the backbone of the road transport network, and transport hubs are the combination of routes and routes, and are the hubs where various transport routes are linked to form a network.

The fuzzy graph model can therefore be used to simulate the road transportation network. Under this circumstance, the broad and flat routes are fuzzy strong cut edges in the fuzzy graph. Fuzzy strong cut vertices in that fuzzy graph model are the key transport hubs in this road transportation network. Predictive maintenance of such routes and key hubs can effectively guarantee the stability of the transportation network.

**Figure 4:**
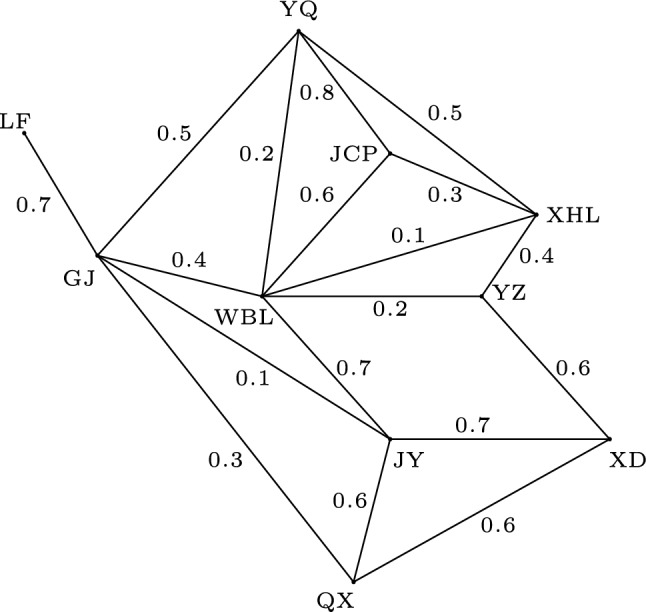
A fuzzy graph *G*.

Taiyuan is a city in China that contains 10 counties. As a transportation network, watermelons from these 10 counties are dispatched to each other. However, different counties have different storage capacities, and there are differences in road transportation capacities between counties. Therefore, it can be modeled by using a fuzzy graph *G* as shown in Fig. 4. As can be seen, the vertices are marked with county abbreviations, i.e. WBL for Wanbailin, XD for Xiaodian, YZ for Yingze, XHL for Xinghualing, JCP for Jiancaoping, JY for Jinyuan, QX for Qingxu, YQ for Yangqu, LF for Loufan, GJ for Gujiao, and the membership values of the vertices denote the storage capacity of watermelons in the county, whilst the membership values of the edges denote the transportation capacity of watermelons by road transport routes between adjacent counties.

In order to keep watermelons fresh, melon farmers need to send watermelons as soon as possible during the ripening period from their place of origin, Qingxu, to Yangqu, where they are mainly sold. It is not difficult to realize that the path QX$\rightarrow$ JY$\rightarrow$ WBL$\rightarrow$ JCP$\rightarrow$ YQ is a unique fuzzy strongest strong path *P* with the strength $s(P)=0.6$ from QX to YQ in the fuzzy graph *G*. Corresponding to the road transportation network, this is a major arterial route. Since deleting any vertex or edge on this path *P* in *G* will result in a decrease in the strength of connectedness between QX and YQ, these vertices JY, WBL, JCP are fuzzy strong cut vertices of *G* and these edges (QX, JY), (JY, WBL), (WBL, JCP), (JCP, YQ) are fuzzy strong cut edges of *G*. Thus, as the person in charge of the transportation network, they should pay close attention to the key transport hubs Jinyuan, Wanbailin, Jiancaoping, and those key routes.

## Conclusion

In an era of increasing technological progress, the safe, reliable and efficient operation of transportation networks is of great significance to economic growth and social development. The establishment of a stable transportation network is a necessary prerequisite to ensure uninterrupted logistics. This paper focuses on the characterization of fuzzy strong cut vertices and fuzzy strong cut edges. Through the analysis, this paper tries to identify the key hubs and routes that support the stability of the network and provide a more systematic and effective method for assessing the stability of the network.

## Data Availability

All data generated or analysed during this study are included in this published article.
